# What device would be best for early infant male circumcision in east and southern Africa? Provider experiences and opinions with three different devices in Kenya

**DOI:** 10.1371/journal.pone.0171445

**Published:** 2017-02-14

**Authors:** Robert C. Bailey, Irene Nyaboke, Fredrick O. Otieno

**Affiliations:** 1 Division of Epidemiology and Biostatistics, School of Public Health, University of Illinois at Chicago, Chicago, Illinois, United States of America; 2 Nyanza Reproductive Health Society, Kisumu, Kenya; University of Pittsburgh, UNITED STATES

## Abstract

Voluntary medical male circumcision (VMMC) reduces risk of HIV acquisition in heterosexual men by approximately 60%. As some countries approach targets for proportions of adolescents and adults circumcised, some are considering early infant male circumcision (EIMC) as a means to achieve sustainability of VMMC for long term reduction of HIV incidence. Evaluations of specialized devices for EIMC are important to provide programs with information required to make informed decisions about how to design safe, effective EIMC programs. We provide assessments by 11 providers with experience in Kenya employing all three of the devices most likely to be considered by various EIMC programs in east and Southern Africa. There was no one device that was seen to be clearly superior to the others. Each had its own advantages and disadvantages. Provider preferences were situation-specific. Most preferred the Mogen Clamp if they themselves were performing the procedure. However, most were concerned that not everyone will have the skills necessary for optimal safety. If someone else were circumcising their son, most would opt for the AccuCirc because of the risk of severing the glans when using the Mogen. A minority preferred the PrePex, but only if the baby received local anesthesia, not EMLA cream (a eutectic mixture of lidocaine 2.5% and prilocaine 2.5%), as presently prescribed by the manufacturer. In the context of a national EIMC program, all participants agreed that AccuCirc would be the device they would recommend due to protection of the glans from laceration and to the provision of a pre-assembled sterile kit that overcomes the need for additional supplies or autoclaving. All agreed that scaling up EIMC, integrating it with existing maternal child health services, will face significant challenges, not least of which is persuading already over-burdened providers to take on additional workload. These results will be useful to programmers considering introduction of EIMC services in sub-Saharan African settings.

## Background

Male circumcision (MC) is a proven HIV prevention intervention, reducing the risk of heterosexual acquisition of HIV in men by 57–67% in three randomized controlled trials and in long-term follow-up studies [1–6]. The World Health Organization (WHO) and UNAIDS approved scale-up of adolescent and adult voluntary medical male circumcision (VMMC) as part of comprehensive HIV prevention programs, and approximately 11.7 million circumcisions have been achieved in 14 east and Southern African countries through 2015 [7]. As a few countries, including Kenya, have achieved their original targets for total VMMCs and as others are expecting to do so in the next few years, governments and donor agencies are considering whether and how best to transition from focusing on adolescent and adult circumcision to early infant male circumcision (EIMC) [8–10].

In 2010, the WHO published the “Manual for Early Infant Male Circumcision under Local Anaesthesia [11],” which included pre-qualification of the Mogen, Gomco and Plastibell devices for EIMC. Since that time, several studies and demonstration projects have employed the Mogen clamp [9,10,12–14], and the Mogen is the device currently approved by the Kenyan Ministry of Health for EIMC [15]. However, other devices are being field tested and are under consideration for adoption in several African countries. These include the AccuCirc [16–18] and most recently the PrePex. These three devices differ in important ways and each has advantages and disadvantages. The Mogen is made of stainless steel and appropriate for repeated uses. It has two flat blades approximately 2.5mm apart through which the foreskin is placed following the angle of the corona, ensuring the glans is not trapped. The blades are clamped together, crushing the tissue and achieving hemostasis. The foreskin is excised along the distal edge of the clamp. The AccuCirc is a single use disposable device designed to protect the glans from laceration or amputation, a possible complication seen in some Mogen cases [19–22]. The device consists of a shielding ring and a single-action clamp that contains a circular blade. The clamp is applied and activated to deliver a circumferential, hemostatic crush while simultaneously incising the foreskin. The PrePex is an elastic compression device with a plastic ring that is placed around the shaft of the penis inside the foreskin proximal to the corona. An elastic compression ring is placed outside the foreskin fitting into the groove of the inner ring. The device is left in situ, the foreskin necrotizes, and is sloughed, while the device detaches spontaneously after a mean of 6 days [23].

Evaluations of specialized devices for EIMC are important to provide programs with the information required to make informed decisions about which devices fit different local needs. We here provide assessments by providers with experience employing all three of the devices most likely to be considered by various EIMC programs in east and Southern Africa. The providers compare the advantages and disadvantages of each device and give their informed opinions on which device would be best to use in different contexts, including as part of a large scale national EIMC program.

## Methods

This study was a discussion group conducted as part of a larger evaluation of the safety and acceptability of the AccuCirc device for EIMC. The participants were clinical officers, nurses, interviewers and mobilizers who had experience in provision of EIMC services with at least two of the three EIMC devices (i.e., AccuCirc, Mogen or PrePex). The two interviewers and a mobilizer were included to gain their insights regarding cultural preferences, demand creation and mothers’ opinions about the procedure. The study participants were all employees of the Nyanza Reproductive Health Society, a Kenyan NGO that has been providing VMMC services since 2007 and EIMC since 2010. The participants did not receive any direct payment for participation but were provided with dinner after the discussion. Participation was voluntary and all participants provided verbal informed consent. Signed informed consent was not required since it would unnecessarily expose the participants to risk of loss of anonymity. An information document was provided to each participant explaining the risks and benefits of study participation and including contact information for the P.I. and the two IRBs. The study was approved by the Maseno University Ethics and Research Committee and the University of Illinois at Chicago Institutional Review Board.

### Study procedures

After providing consent, participants were asked to fill out a brief anonymous questionnaire to record their age, marital status, education, and cadre (clinical officer, nurse, research assistant, mobilizer), number of male children, whether their son or sons were circumcised, and approximate number of procedures performed with each device. A discussion guide in English was developed based on the literature and our previous studies and covered the following topical areas: familiarity and experiences with EIMC devices, challenges using each device, advantages of each device, preferences for devices under different scenarios, and barriers and facilitators for scaling up EIMC. All participants had greater than a secondary school education and were fluent in English.

The discussion, which lasted approximately two hours, was led by an experienced moderator, and a note taker was present to record the discussion nearly verbatim. In addition, the discussion was audio taped with the participants’ permission. The notes were typed within 48 hours and checked against the audio-tapes, with any discrepancies corrected. Transcripts were coded using Nvivo Version 8 (QSR International, Australia) with codes developed from the topics and probes targeted in the discussion guide. A few additional codes were developed from points that emerged after a preliminary reading of the transcripts.

## Results

### Participant characteristics

The study included 11 participants: 6 (55%) women and 5 (45%) men (Table 1). The median age was 32 years (range 24–45). Three participants were clinical officers, five were nurses, two were interviewers, and one was a mobilizer. All eight clinicians had performed EIMC using both the Mogen clamp and the AccuCirc, while five of the clinicians also had experience with the PrePex device.

**Table 1:** Characteristics of study participants.

Characteristic	Number	Percent
Sex		
Male	5	46
Female	6	54
Age		
Median	32	
Range	24–45	
Education		
Diploma in Clinical Medicine	3	27
Other Diploma	3	27
Bachelors of Nursing	4	36
Other Bachelors	1	9
Designation		
Clinical Officer	3	27
Nurse	5	45
Research Assistant	2	18
Mobilizer	1	9
EIMC Procedures by Device		
Mogen	2118	76
AccuCirc	752	26
PrePex	66	2
For those with sons, are they circumcised?		
Yes	5	100
No	0	0

### Knowledge of available EIMC devices

Participants were asked to name devices and techniques used for EIMC. In addition to the three devices that were the focus of the discussion, others that were mentioned were: the Gomco Clamp, Plastibel and Shang Ring. Of these, the Shang Ring has not been approved for EIMC. Free hand and dorsal slit surgical methods were also mentioned.

### Challenges and advantages of using the Mogen, PrePex and AccuCirc

Participants were asked their opinions and experiences with each of the devices and to describe the advantages and challenges of using each.

#### Advantages and challenges using the Mogen clamp

The advantages to using the Mogen clamp that participants mentioned included that the procedure was fastest using the Mogen. Most participants agreed that it was very efficient at achieving hemostasis. As one participant said, “It never fails to deliver the pressure that is required to control bleeding.” Compared to the other devices, participants felt that adverse events, especially cases of bleeding, were fewer using the Mogen Clamp. Another advantage mentioned was that it is the easiest device to learn to use and the easiest to train providers to use.

The biggest challenge in using the Mogen that all providers agreed upon was the risk of injuring or severing the glans penis. As one participant said, *“There is a fear of the unknown when opening the device after cutting*. *There is anxiety to open up the clamp and check whether the glans has been severed*. *For smaller infants*, *as you pinch the foreskin to slide in the device*, *it is difficult to feel whether the glans is out of the way*.*”*

Several participants mentioned that the risk of a severed glans may be increased if the angle of the opening of the clamp is larger than it should be and that clamps should be checked regularly, since the opening can be enlarged by repeated autoclaving.

#### Advantages and challenges using the AccuCirc

The main advantage of using the AccuCirc device that participants cited was that there was no risk of injuring the glans. A typical statement was:

“*Accucirc is 100% safe*. *There are no worries about severing the glans*.”

The other advantage mentioned was that the AccuCirc comes in a complete sterile package with everything needed for the procedure. This was seen as ideal for use in situations where autoclaving and additional instruments may not be available. Participants considered this to be convenient and could be cost-saving.

Nearly all participants cited the greatest disadvantage to using the AccuCirc as the risk of the device not achieving a complete cut of the foreskin. This is fairly easily addressed with scissors by simply completing the cut; however, some incomplete cuts result in bleeding, and participants were concerned that bleeding is an adverse event that occurs more often with the AccuCirc than the other devices.

“*After inserting the ring you are not sure if there will be an incomplete cut or bleeding*. *Sometimes bleeding starts during the 1 hour post procedure observation period*. *One is not confident that they are sending a child home and he won’t bleed*.”

It was pointed out that when the AccuCirc is used, there should be a clinician available who has experience in suturing. This is necessary in the event that the application of pressure to a bleeding wound does not staunch the bleeding. Someone mentioned that such suturing skills should be available no matter the technique used for EIMC, although they may be needed more when the AccuCirc device is used.

“*Suturing skills should cut across [all the devices]*.”

#### Advantages and challenges using the PrePex device

The participants cited several advantages of using the PrePex device for EIMC. They felt that the cosmetic outcome was very good. They also felt that it was easy to learn to use and that it would be easy to train others. This is primarily because application of the PrePex device does not require suturing skills. However, all expressed concern that placement of the inner and outer rings was difficult and required a lot of practice. Some participants said that once the device is in place, mothers expressed fewer concerns than with other techniques; however, others said that with PrePex mothers called a lot with questions and concerns during the one-week period between placement and detachment of the device.

A few participants pointed out that the PrePex had not been used previously for EIMC except in Rwanda and that there were aspects of its use that still required further refinement. One of these was the application of EMLA to achieve anesthesis. There was the question of how best to apply the EMLA. But of more concern to all the clinicians was that the EMLA did not achieve the level of anesthesis required for the comfort of the infant, and the levels of pain were very troubling.

“*If you read the anatomy of innervation for the penis*, *you will see that EMLA only numbs the ventral side and the dorsal nerve is not touched*.”

“*The biggest challenge I faced was pain*, *especially when removing adhesions*. *I would say EMLA doesn’t work*.”

“*Lignocaine injection would be better because with PrePex*, *babies cry a lot in pain*.”

It was the consensus that a dorsal block similar to that used with the other two techniques was as necessary when using the PrePex as when using the other devices.

The participants spent quite a lot of time expressing their concerns about placing the inner ring of the PrePex device correctly so that it interfaces with the outer placement ring. Some were still experimenting with how to best achieve proper placement.

“*During training we were told to insert the ring perpendicularly*, *but I found a different way where the ring approaches the glans horizontally*. *It requires practice so you get better with time*.”

Some discussed the difficulties of placing the inner ring with different penile and foreskin sizes:

“*If you get a short shaft and a long foreskin*, *placing the ring is quite hard*.”

“*Another challenge is a narrow foreskin*. *Makes it hard to insert the ring and the skin might tear*. *With the narrow foreskin it is also difficult to view whether ring is in place so there is a fear of invagination*.”

“*If the skin tears or you have a hard time placing the ring*, *you might have to resort to using a Mogen. We have had three such cases [out of a total of 50 cases]*.”

### Which device do you prefer?

The participants were asked to weigh the challenges and advantages of each EIMC method and to express their preference under three different scenarios: First, if they themselves are performing the procedure; second, if a different provider is circumcising their infant; and third, if EIMC is scaled up in Kenya and throughout east Africa.

#### Which method do you, as a provider, prefer to use and what are the reasons?

Five of the 11 participants expressed their preference for the Mogen. The reasons they gave included the reduced chances of bleeding, reduced pain due to local anesthesia, and speed and ease of the procedure. One participant said,

“*I believe the foreskin should be removed instantly*, *so [I prefer] the Mogen*.”

Three participants expressed a preference for the PrePex, saying that the pain is brief and the cosmetic outcome is good. Two participants said that they would prefer the PrePex, but only if local anesthesia was administered. Two participants said their choice would depend on the age and size of the baby.

“*It depends on age*. *Up to 30 days I would prefer AccuCirc*. *For greater than 30 days I would prefer Mogen*.”

“*AccuCirc and PrePex*, *depending on what I see on the baby*. *If the baby is bigger and parent is ok with a non-surgical method*, *then I would use PrePex*. *Otherwise I would use AccucCirc because of safety*.”

These participants were concerned that the risk of incomplete cuts and bleeding with the AccuCirc was greater with larger, older babies.

One participant said that he had no preference; he was fine with using any of the three methods.

#### If you have an infant boy, which device would you prefer to be used by another provider to circumcise your son?

Under these conditions, most of the participants expressed preference for the AccuCirc.

“*Accucirc because there is 100% surety of not severing the glans*. *The circumcision cut is achieved instantly*. *There are no unknowns like odor with PrePex*.”

“*Accucirc because of safety*, *and also because of my experience*. *My son was circumcised with Mogen and I was very worried about severing the glans*.”

“*Accucirc because of safety of the glans*.”

Two participants said that they would prefer the use of Mogen, but only if they knew and trusted the provider; otherwise they would opt for the AccuCirc:

“*Mogen*, *if done by a trusted colleague*. *Accucirc if there is no trusted health care worker available because of safety*.”

“*All my sons have had surgical circumcisions*. *I would go for Mogen if I trust the provider*. *If I was not a clinician*, *I would go for AccuCirc because any adverse events are manageable and don’t have far reaching consequences*.”

“*Mogen*, *but I would ensure it is an experienced provider*.”

Only one person preferred that her son be circumcised using the PrePex device.

“*My son was circumcised with Mogen and he cried [for hours]*. *I would go for PrePex*. *Mums all say that the baby does not cry once the device is placed*.”

#### Which device should be used if EIMC is scaled up in Kenya?

The study participants were unanimous in their preference for AccuCirc as the device to be chosen in the event of a national scale-up of EIMC. The major reasons were due to its safety, ease of operation, and costs in terms of human resources needed.

“*AccuCirc because in many health facilities there is a shortage of staff*. *It needs only one operator*. *PrePex may need two*. *Also safety reasons*, *since it is safer than Mogen*. *Also*, *logistics for sterilization in public facilities will be easier to manage with the AccuCirc kit*, *which comes complete*”

“*If there is no follow up, Prepex device is locked out*. *I would go for Accucirc because it is cheaper in terms of human resources*.”

“*AccuCirc because of workload*. *If babies are many*, *health care workers might be exhausted*, *and chances of severing glans are eliminated*. *AccuCirc is easier with smaller babies*.”

### If Kenya were to scale-up EIMC, what might be some of the challenges to achieve a successful program?

Participants had many insights into possible barriers and ways in which some of them might be overcome. Their responses can be divided into three different areas: community factors, parental factors, and provider/facility factors.

*Regarding community factors*, it was pointed out that EIMC is little known or understood in Kenya, among both communities where circumcision is traditionally practiced and where it is not traditionally practiced. The consensus was that it would take time for EIMC to be widely adopted by either type of community.

“*Some communities want to circumcise later when it is considered a rite of passage*. *Non circumcising communities find it suspicious*. ‘*Why are they forcing us to do what our forefathers did not do*?’ *This brings about identity issues*.”

“*The uncircumcised adults do not allow their infants to be circumcised*. *As more men get circumcised*, *EIMC may be more acceptable*.”

“*The circumcised boy (for now) is the anomaly*, *not the norm*.”

“*The current group of infants [who are getting circumcised]*, *once they reach nursery*, *it will be more the norm*.”

*Regarding parental factors*, the participants felt that concerns about pain and safety were especially salient for mothers.

“*Perceived pain by the parents*. *Parents feel that babies are still too small and need to rest*. *There is the perception that the child is delicate and should not be subjected to such trauma*.”

Another factor mentioned was whether the father or an older brother has been circumcised. Most mothers will not make the decision to circumcise their baby without the father’s approval. This makes recruitment and consenting more difficult than for adult VMMC, and it often places the decision in the hands of the father.

“*Fathers who have not been circumcised will not agree to have their son circumcised*.”

“*Maybe the firstborn child was not circumcised and [the] mother does not want the younger son to be circumcised before the older one*.”

Certain religious and cultural practices were mentioned as barriers to adoption. For example, in rural communities, mothers are not permitted to observe the penis of their boy child until after their post-partum vaginal discharge (lochia) has ceased. This can be anywhere from ten days to two months after the birth, requiring someone else—a sister or other family member—to bring the baby for the procedure.

“*Also*, *some churches do not allow babies to get out of the house until after 2 months*.”

Another participant mentioned that accessibility and cost will be factors contributing to uptake, and he pointed out that some parents seek the service in private clinics where it is expensive. And another said that many parents want more information about EIMC.

“*We need education and demand creation*.”

*Regarding provider and facility level factors* that should be taken into consideration if Kenya is to scale up EIMC, all the participants expressed doubt that staff in health facilities would take on the additional burden of providing EIMC unless they received incentives. This is in part because the history of introducing new services in health facilities—especially HIV prevention, care and treatment services—has included either additional remuneration or additional staffing to cover the new service. Another method of introducing additional services has been through parallel programs not integrated with existing public health services and implemented by partner organizations that hire and pay their own staff and receive separate donor funding.

“*The perception of providers is that there are donor funds that should trickle down to them which is a challenge scaling up VMMC*, *and it will be the same with EIMC*.”

“*They view the program as a cash cow*. *They expect cash since they don’t do it routinely*.”

“*We trained over 20 healthcare workers in facilities to do EIMC*, *but none are currently offering the service in the facilities*.”

The participants discussed possible solutions to these challenges and felt that it would take strong leadership from the Ministry of Health to integrate EIMC with maternal, neonatal child health (MNCH) services. If there were no additional funds to pay staff for adding EIMC to their duties, then achieving significant numbers of infant circumcisions will be challenging. However, the MOH could make EIMC a mandatory, reportable service and require facilities to meet targets.

“*To make EIMCs to be part of the reportable data to the MOH might push the staff to do more procedures*.”

Some other comments that participants had regarding scaling up EIMC nationally included concerns about getting informed consent from parents.

“*Some mothers are willing to give consent for the procedure*, *but most need to consult the father of the baby*. *This can make recruitment more complicated and time-consuming than for VMMC*.”

There was concern about safety on several levels. If providers do not do large numbers of EIMCs in some facilities, their skills may not be maintained, and there could be more adverse events than seen so far in research settings. One participant suggested that this problem may be especially acute in rural settings since uptake will likely be lower in rural areas—just the areas farthest from back-up emergency facilities. Another mentioned that diagnoses of signs and symptoms that make adult men ineligible for VMMC are more difficult to detect in infants.

“*We take a history early in life and may miss out on underlying congenital anomalies*. *With adults there is a clear history*, *making their screening easier*.”

## Discussion

As policymakers and donors consider whether and how to transition to sustainable models of male circumcision for HIV prevention, some programs are assessing the feasibility and safety of introducing EIMC services into health facilities as part of MNCH programs [8–10]. Knowing the attributes and challenges associated with different EIMC methods will be critical to making informed choices regarding best and safest EIMC practices. This is the only study to date that reports the opinions of healthcare providers who have experienced provision of EIMC services using three different infant circumcision devices: the Mogen Clamp, the PrePex device, and the AccuCirc device. Their informed opinions will be useful to programmers considering introduction of EIMC services in sub-Saharan African settings.

There was no one device that was seen to be clearly superior to the others. Each had its own advantages and disadvantages. Notably, provider preferences were situation-specific. Most preferred the Mogen if they themselves were performing the procedure. However, most were concerned that not everyone will have the skills necessary for optimal safety. When asked which device they would recommend if someone else were circumcising their son, most would opt for the AccuCirc because of the risk of severing the glans when using the Mogen. A minority preferred the PrePex, but only if the baby received local anesthesia, not EMLA cream (a eutectic mixture of lidocaine 2.5% and prilocaine 2.5%), as presently prescribed by the manufacturer. In the context of a national EIMC program, all participants agreed that AccuCirc would be the device they would recommend due to protection of the glans from laceration and to the provision of a pre-assembled sterile kit that overcomes the need for additional supplies or autoclaving. They also all agreed that scaling up EIMC, integrating it with existing MNCH services, will face many challenges, not least of which being to persuade providers to take on an additional workload when they are already overloaded and poorly compensated.

WHO has pre-qualified three devices for EIMC: the Mogen, Gomco and Plastibell devices [24]. The Gomco clamp has an excellent safety record as a stainless steel bell protects the glans from the risk of laceration or amputation. However, a limitation of this clamp is that it requires four different parts and exists in many different sizes which require the provider to use the correctly sized parts when assembling the device [11]. As far as we are aware, there have been no evaluations of the Gomco or Plastibell in Kenya. A trial comparing the Mogen clamp to the Plastibell in Botswana found that minor adverse events were more common with the Mogen compared with the Plastibell; however, there were two severe adverse events with the Plastibell. The authors expressed preference for the Mogen clamp due to the risk of migration and retention of the Plastibell, which could result in necrosis of the glans in the absence of the kind of follow-up inherent to a research study [25]. The Mogen is the device currently approved by the Kenya Ministry of Health for EIMC [8], and several studies and demonstration projects have chosen to employ the Mogen clamp [12–14, 17, 18], including two studies conducted by our group [12, 19, 26, 27]. Adverse event rates when employing the Mogen are relatively low. However, when an adverse event occurs, it can be a laceration to the glans or even an amputation. Such events are rare, but they do occur [19–22, 27], and all providers using the Mogen are very fearful that they may inadvertently cause damage no matter how much care they take. As one provider stated it, *“When I close that clamp*, *my heart is in my throat and I am praying that I have not pinched the glans*.*”*

The AccuCirc device avoids any risk of damage to the glans. It has undergone evaluation in a single-arm study in Botswana with no serious adverse events [16]. A randomized non-inferiority trial of the AccuCirc versus the Mogen conducted in Zimbabwe found no difference in AE rates when employing the two devices [17], and a subsequent study by the same group found that AE rates were low (1.4%) among 500 circumcisions performed by nurse-midwives using the AccuCirc, with no serious AEs (SAE). In Kenya, among 600 EIMCs performed by our group with the AccuCirc, we had an AE rate of 2.8% with no SAEs [18]. An analysis of costs of EIMC found that AccuCirc has lower unit cost ($49.53) versus the Mogen ($55.93), primarily due to lower costs of consumable supplies [28]. The AccuCirc procedure has been found to be acceptable to parents and could increase uptake of EIMC [16–18]. The drawback of the AccuCirc is the high frequency of incomplete or partial cuts [16,18] in which the blade does not cut completely through the entire circumference of foreskin, requiring completion of the incision with scissors. Some incomplete cuts can result in bleeding that requires one or a few sutures [16,18].

The participants in our study expressed concern that integrating EIMC services with ongoing reproductive, maternal, newborn and child health platforms will be challenging primarily because of human resource constraints, competing priorities and desire for compensation for additional responsibilities. Such challenges have been voiced in the context of incipient programs in other countries [9] and in our experimental programs in Kenya [28]. These, along with the need for large scale training in the face of limited initial demand for EIMC, are challenges that will require innovation and collaboration across all segments of the healthcare system to address.

The primary limitation of this study is its small sample size of just 11 EIMC providers. As more doctors, clinical officers and nurses become trained and gain EIMC experience using different devices, the views regarding methods and how to scale up EIMC services safely and efficiently may change. This may be especially true once there is more experience with the PrePex device. However, at this time, we are unaware of any other study that has included providers who have experienced the use of the three main EIMC devices likely to be considered for scale-up of national EIMC programs in east and southern Africa. Thus this report should prove very useful to many programmers, donors and practitioners as they design their EIMC programs. Also, this study is novel for asking providers their opinions about which device is preferable under varying circumstances, going beyond preference for personal use to include preference for which device would be best in a national scale-up scenario.

## Conclusion

Based on the experience and preferences of the providers included in this study who have performed EIMCs using three different devices, the AccuCirc device may be the safest and most preferred device for use in a large-scale regional or national EIMC program. The process for bringing the AccuCirc device to the WHO pre-qualification stage should move forward as countries consider implementation of sustainable circumcision programs for comprehensive HIV prevention. In addition, this study highlights some of the many challenges that programmers will face as they seek to initiate EIMC in east and southern Africa. Among those mentioned in this study are: lack of familiarity with EIMC on the part of both populations and providers, cultural and religious barriers, demand creation, low initial uptake, obtaining parental consent, comprehensive screening of infants, safety of the procedure itself, emergency response capability, training of providers, ensuring maintenance of provider skills, and integration with maternal neonatal child health services.
